# Aryl-functionalised α,α′-Trehalose 6,6′-Glycolipid Induces Mincle-independent Pyroptotic Cell Death

**DOI:** 10.1007/s10753-023-01814-5

**Published:** 2023-05-04

**Authors:** Kristel Kodar, Emma M. Dangerfield, Amy J. Foster, Devlin Forsythe, Shigenari Ishizuka, Melanie J. McConnell, Sho Yamasaki, Mattie S. M. Timmer, Bridget L. Stocker

**Affiliations:** 1 School of Chemical and Physical Sciences, Victoria University of Wellington, PO Box 600, Wellington, New Zealand; 2 Centre for Biodiscovery, Victoria University of Wellington, PO Box 600, Wellington, New Zealand; 3 School of Biological Sciences, PO Box 600, Wellington, New Zealand; 4 Department of Molecular Immunology, Research Institute for Microbial Diseases, Osaka University, Suita, Osaka Japan; 5 Laboratory of Molecular Immunology, Immunology Frontier Research Center, Osaka University, Suita, Osaka Japan; 6 Division of Molecular Immunology, Medical Institute of Bioregulation, Kyushu University, Fukuoka, Fukuoka Japan; 7 Division of Molecular Immunology, Medical Mycology Research Center, Chiba University, Chiba, Japan

**Keywords:** mincle, pyroptosis, macrophage, adjuvant, inflammasome, Caspase-1.

## Abstract

α,α′-Trehalose 6,6′-glycolipids have long been known for their immunostimulatory properties. The adjuvanticity of α,α′-trehalose 6,6′-glycolipids is mediated by signalling through the macrophage inducible C-type lectin (Mincle) and the induction of an inflammatory response. Herein, we present an aryl-functionalised trehalose glycolipid, AF-2, that leads to the release of cytokines and chemokines, including IL-6, MIP-2 and TNF-α, in a Mincle-dependent manner. Furthermore, plate-coated AF-2 also leads to the Mincle-independent production of IL-1β, which is unprecedented for this class of glycolipid. Upon investigation into the mode of action of plate-coated AF-2, it was observed that the treatment of WT and Mincle^−/−^ bone marrow derived macrophages (BMDM), murine RAW264.7 cells, and human monocytes with AF-2 led to lytic cell death, as evidenced using Sytox Green and lactate dehydrogenase assays, and confocal and scanning electron microscopy. The requirement for functional Gasdermin D and Caspase-1 for IL-1β production and cell death by AF-2 confirmed pyroptosis as the mode of action of AF-2. The inhibition of NLRP3 and K^+^ efflux reduced AF-2 mediated IL-1β production and cell death, and allowed us to conclude that AF-2 leads to Capase-1 dependent NLRP3 inflammasome-mediated cell death. The unique mode of action of plate-coated AF-2 was surprising and highlights how the physical presentation of Mincle ligands can lead to dramatically different immunological outcomes.

**Supplementary Information:**

The online version contains supplementary material available at 10.1007/s10753-023-01814-5.

## INTRODUCTION

**Fig. 1:**
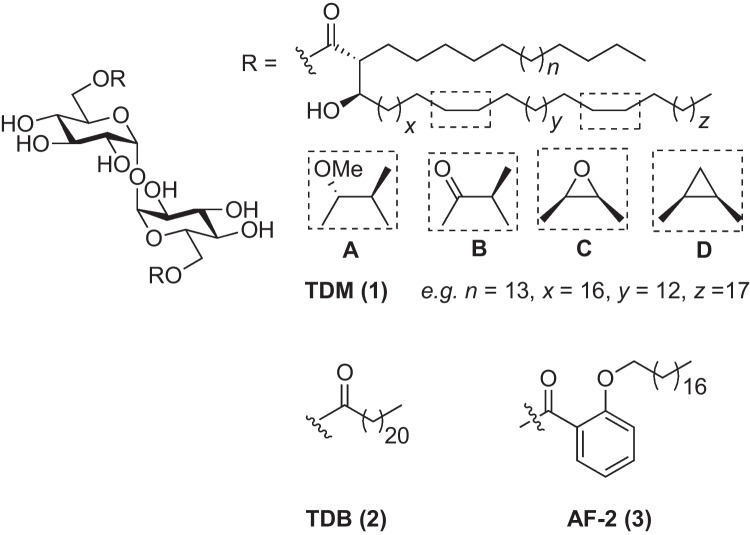
α,α′-Trehalose 6,6′-glycolipids TDM (**1**), TDB (**2**) and AF-2 (**3**).

Trehalose glycolipids containing an α,α′-trehalose core and lipophilic acyl chains at the 6- and 6′-positions have long been known for their immunomodulatory activity [1–3]. Trehalose dimycolates (TDMs, **1**, Fig. 1), the major cell wall component of *Mycobacterium tuberculosis*, was first identified as an important virulence factor of mycobacteria in the 1950s [4]. Some twenty years later, the glycolipid, and derivatives thereof, were found to exhibit promising anti-cancer and anti-bacterial activity [1]. In 2005, trehalose 6,6′-dibehenate (TDB, **2**) demonstrated powerful adjuvanticity when combined with the quaternary ammonium compound dimethyldioctadecylammonium (DDA) [5], and this formulation has since been used in the development of several vaccines [6]. Although it has been known for some time that α,α′-trehalose 6,6′-glycolipids can induce a pro-inflammatory immune response, it was not until 2009 that TDM (**1**) and TDB (**2**) were found to exhibit their inflammatory response by signalling through the macrophage inducible C-type lectin (Mincle, Clec4e, or Clecsf9) [7–9], a pattern recognition receptor that is expressed on the surface of innate immune cells such as macrophages, dendritic cells (DCs), and neutrophils [10, 11]. TDB and TDM can simultaneously provide a priming signal and an inflammasome-activating signal [9, 12], with Mincle binding by TDB or TDM being a prerequisite for the induction of the FcRγ-Syk-Card9 signalling axis, NF-κB activation, and the production of pro-inflammatory cytokines [2, 7–9, 13–15].

Given the immunostimulatory activity of α,α′-trehalose 6,6′-glycolipids, we, and others, have been interested in exploring how these compounds can be modified or formulated to enhance their therapeutic potential [1–3]. Studies include how the solubility of α,α′-trehalose 6,6′-glycolipids affects the compound’s ability to induce an inflammatory response [16, 17], how the glycolipids can alter macrophage phenotype [18] and activate dendritic cells or T-cells for use as anti-cancer therapies [19], or be incorporated in self-adjuvanting anti-cancer vaccines [20]. Screening of libraries of α,α′-trehalose 6,6′-glycolipids has been a popular approach for the identification of derivatives with promising adjuvanticity, particularly within the context of vaccines against infectious disease [2, 21–25]. When investigated, the inflammatory or anti-cancer response of the α,α′-trehalose 6,6′-glycolipids has been found to be Mincle-dependent.

As part of our on-going studies into the use of glycolipids as vaccine adjuvants, we were interested in exploring the adjuvanticity of a series of aryl-functionalised α,α′-trehalose 6,6′-glycolipids. Studies by ourselves [21, 26] and Evans *et al.* [23, 24] provided evidence for the promising adjuvanticity of this class of compounds and led us to screen further aryl α,α′-trehalose 6,6′-glycolipids for their immunomodulatory activity [25, 27]. Insomuch, one particular α,α′-trehalose 6,6′-glycolipid, AF-2 (**3**), induced strong Mincle signalling, as determined using NFAT-GFP reporter cells, and high levels of IL-1β by bone marrow derived macrophages (BMDMs) [25]. We reasoned that the immunostimulatory activity of AF-2 would be Mincle-dependent, as was observed for all previously studied α,α′-trehalose 6,6′-glycolipids. Herein, we sought to confirm the mode of action of plate-coated AF-2 and unexpectedly observed the Mincle-independent production of IL-1β. Further analysis revealed that plate-coated AF-2 led to capase-1 dependent NLRP3 inflammasome-mediated pyroptotic cell death.

## MATERIALS AND METHODS

### Preparation of AF-2 and TDB

AF-2 [25] and TDB [28] were prepared according to previously published protocols and determined to be endotoxin free (≤ 0.1 EU/mL) using the Pierce^™^ LAL Chromogenic Endotoxin Quantitation Kit (Thermo Scientific).

### Animals

C57BL/6 WT mice and Mincle^−/−^ mice were bred and housed in a conventional animal facility at the Malaghan Institute of Medical Research, Wellington, New Zealand, or Osaka University, Japan. All animals used for the experiments were aged between 8–12 weeks.

### Murine Bone Marrow-Derived Macrophage Assay with Plate-Bound Ligands

α,α′-Trehalose 6,6′-glycolipids, AF-2 and TDB, were made up to 1 mM in CHCl_3_/MeOH (2/1, v/v), then diluted to 400 μM or 50 μM in isopropanol to give 8 or 1 nmol of glycolipid per 20 μL, respectively. The solutions were then added to 96-well plates (20 µL/well) and the solvent was evaporated within a sterile hood for 18 h.

Bone marrow cells were collected from the tibia and femur of C57BL/6 or Mincle^−/−^ mice and cultured (250,000 cells/mL) in complete Roswell Park Memorial Institute medium [cRPMI-1640 (Gibco) with 10% heat inactivated fetal bovine serum (Gibco), 100 unit/mL penicillin-streptomycin (Gibco) and 2 mM Glutamax (Gibco)]. Macrophage differentiation was induced by 50 ng/mL GM-CSF (PeproTech) added to the cRPMI. Cells were incubated at 37 °C (5% CO_2_) for 8 days (cells fed on days 3 and 6). On day 8, the media was removed, the cells were lifted using Accutase (StemCell), counted and re-seeded in 96-well plates coated with ligands. Where indicated Ac-YVAD-cmk (40 μM, Sigma) or KCl (50 mM) [12] were added to the cells an hour before, or CY-09 (20 or 40 µM) was added 30 min before adding the cells to ligand-coated plate according to the manufacturer’s protocol. 100 ng/mL LPS (Sigma) or nigericin (10 μM, ChemImpex) were used as controls [29].

### Bone Marrow-derived Macrophage Assay with Solubilised Ligands

Bone marrow cells were collected from the tibia and femur of C57BL/6 or Mincle^−/−^ mice and cultured as described above. On day 8, all media together with non-adherent cells were removed and 0.25 mL fresh media was added to each well (48 well plate). Stock solutions of AF-2 and TDB (1 mM, with 2% DMSO in sterile water) were prepared and kept sterile. From these stock solutions, 10 μL were added to each well to give a final ligand concentration of 40 μM in a total well volume of 250 μL. A positive control with 100 ng/mL LPS and a negative control with untreated cells were used. Stimulated cells were incubated for 24 h before the supernatants were analysed for IL-1β production.

### Human Cell Isolation

The use of human leukocytes from healthy donors with written informed consent was approved by New Zealand Northern A Health and Disability Ethics Committee (approval number 15/NTA/178/AM04). Human monocytes were enriched from whole blood by negative selection [30] using RosettaSep Human Monocyte Enrichment Cocktail (StemCell) according to the manufacturer’s instructions. The cell concentration was adjusted to 1 × 10^6^ cell/mL in cRMPI and 100 μL of cells were added per well in a ligand-coated 96-well plate.

### Cytokine Analysis

Levels of murine IL-1β, IL-23, IL-10, MIP-2 (R&D Systems), IL-18 (Sino Biological), IL-13 (Invitrogen), TNF-α, IL-6, IL-12p40, and human IL-1β (BD Biosciences) cytokines in the supernatants were determined by sandwich ELISA according to the manufacturer’s instructions.

### Detection of Reactive Oxygen Species (ROS)

Bone marrow cells from WT and Mincle^−/−^ mice were cultured in 10% FCS/RPMI supplemented with 50 ng/mL GM-CSF (BioLegend) for eight days. Attached cells were detached by 1 mM EDTA-supplemented PBS and were used as BMDMs. The cells were added into 96-well plates coated with 8 nmol/well of AF-2 or TDB, or 100 μg/mL zymosan (SIGMA), in the presence of 300 μM luminol (Nacalai tesque) for 60 min. Luminescence was quantified by POWERSCAN HT (DS Pharma Biomedical). Data are presented as mean ± SE of triplicate assays.

### Western Blot

Wild type and Mincle^−/−^ GM-CSF BMDMs were differentiated over 8 days, lifted using Accutase (StemCell) and stimulated on 96-well plates coated with AF-2 or TDB (8 nmol/well) or nigericin (10 μM, positive control) for 4 h. Whole cell lysate was collected with IGEPAL^®^ CA-630 (Sigma) buffer containing protease (cOmplete Tablets, Roche) inhibitors. Western blots were performed by 12% SDS-PAGE and wet blotting. Antibodies used were anti-GSDMD [EPR19828] (Abcam), anti-β-actin [D6A8], anti-rabbit IgG-HRP (Cell Signaling Technology). Blots were developed with Pierce^™^ ECL Western Blotting Substrate (Thermo Scientific) and imaged with Amersham Imager 600.

### Sytox Green Assay

WT and Mincle^−/−^ GM-CSF BMDMs were differentiated over 8 days and lifted using Accutase (StemCell). Sytox Green (1 μM, Invitrogen) was added to the media before adding the cells to 96-well plates coated with AF-2 or TDB (8 nmol/well). Nigericin (10 μM) was used as a positive control. Fluorescence of Sytox Green was measured at the indicated time points (cells kept in 37 °C, 5% CO_2_ between measuring) using EnSpire plate reader (Perkin Elmer) at 523 nm; results are calculated relative to iPrOH control fluorescence.

### Lactate Dehydrogenase Assay

LDH was measured from supernatant using CyQUANT^™^ LDH Cytotoxicity Assay (Invitrogen). Percentage of cytotoxicity was calculated using spontaneous LDH activity and maximum LDH activity controls according to the manufacturer’s instructions.

### Confocal Microscopy

WT and Mincle^−/−^ GM-CSF BMDMs were differentiated over 8 days, lifted using Accutase (StemCell) and seeded on cover slides coated with iPrOH as a control, 8 nmol/slide AF-2 or TDB, or treated with Nigericin (10 μM) as a control for pyroptosis. After 4 h, the cells were washed with D-PBS (Gibco) and stained with anti-myeloperoxidase (MPO) antibody [2D4] (FITC conjugated, Abcam), anti-histone H4 (acetyl K16) antibody [EPR1004] (Alexa Fluor 647 conjugated, Abcam) and DAPI (BD Pharmagen) for 30 min. After further washing with PBS, ProLong^™^ Diamond Antifade Mountant (Invitrogen) was used to mount cover slips onto microscope slides. Confocal images were taken using the inverted microscope IX83 equipped with a confocal head FV3000, employing the blue filter to observe the DAPI dye, red filter for H4 and the green filter to observe the MPO. The BMMs with media only treatment was used to correct for autofluorescence of the cells.

### Scanning Electron Microscopy

BMDM cells were seeded on conductive silicon wafers, coated with AF-2 (8 nmol/slide) or left untreated (untreated control, staurosporine and nigericin controls), and allowed to adhere overnight. Nigericin/staurosporine control cells were first treated with 1 μg/mL of LPS for 4 h. LPS was removed and the cells were further treated with 10 μM nigericin for 1 h or 5 h with 1 μM staurosporine, respectively. At the end of each treatment the media was removed, and the cells were fixed overnight at 4 °C using Karnovsky’s EM fixative, with paraformaldehyde, glutaraldehyde, calcium chloride and cacodylate buffer, at 3% strength. Fixation buffer was removed, cells rinsed using PBS and dehydrated through a graded series of ethanol (30, 50, 70, 95 and 100%) and acetone (100%) followed by critical point drying with liquid CO_2_. RAW264.7 cells were primed with LPS (1 μg/mL) and seeded on conductive silicon wafers coated with AF-2 or TDB (8 nmol/slide) or treated with staurosporine (1 μM) or with nigericin (10 μM). Following treatment, the cells were fixed overnight at 4 °C using Karnovsky’s EM fixative, rinsed briefly using water and ethanol, and snap frozen using liquid nitrogen to observe morphology using CryoSEM. Dried CryoSEM and SEM specimens were sputter coated with platinum and imaged with a JEOL JSM 6500F field emission scanning electron microscope operating at 20 kV (SEM) or 10 kV (CryoSEM).

### Statistics

Statistical significance of differences was assessed using two tailed 2-way ANOVA with Dunnett’s multiple comparison test or Multiple t-tests where appropriate, using Prism v8 software (GraphPad). A P value less than 0.05 was considered statistically significant.

## RESULTS AND DISCUSSION

### Plate-coated AF-2 Leads to IL-1β Production by BMDMs in a Mincle-dependent and Mincle-independent Manner

**Fig. 2:**
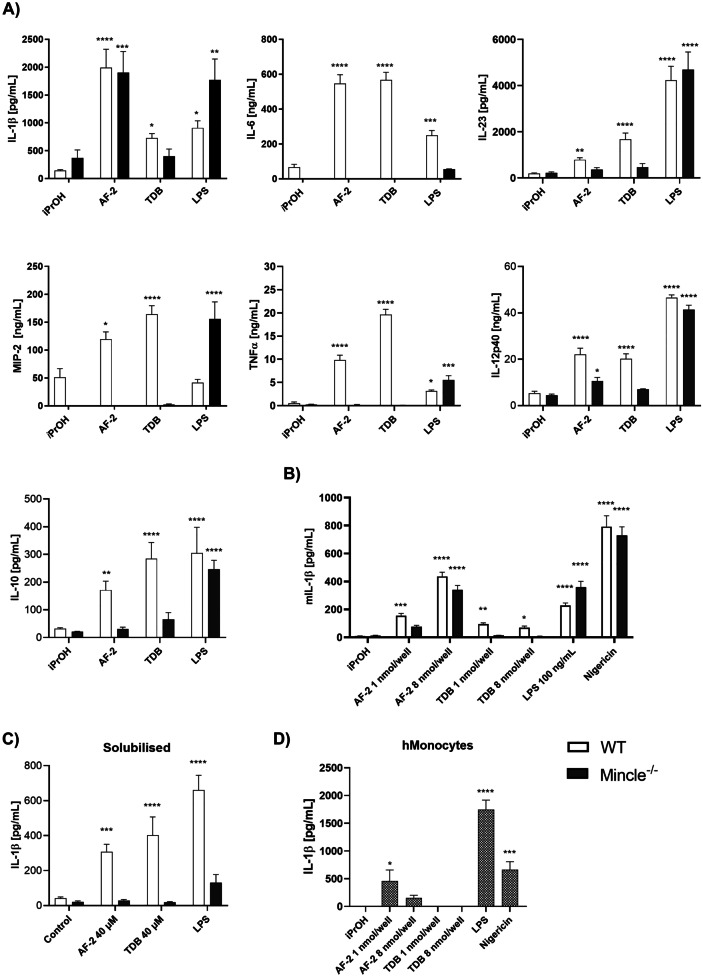
Aryl-trehalose glycolipid AF-2 induces Mincle-independent IL-1β production. **a** WT and Mincle^−/−^ GM-CSF BMDMs and were incubated with plate-bound AF-2 or TDB (1 nmol/well; solubilised LPS (100 ng/mL) as a control) and IL-1β, IL-6, MIP-2, TNF-α, IL-12p40, and IL-10 production measured at 24 h by ELISA. **b** WT and Mincle^−/−^ GM-CSF BMDMs were incubated with plate-bound AF-2 or TDB (1 or 8 nmol/well; solubilised LPS (100 ng/mL) or nigericin (10 μM) as a control) and IL-1β production measured at 24 h by ELISA. **c** WT and Mincle^−/−^ GM-CSF BMDMs were stimulated with AF-2 or TDB (40 μM; solubilized in 2% DMSO in H_2_O) or LPS (100 ng/mL) and IL-1β was measured at 24 h by ELISA. **d** Negatively selected human monocytes were incubated with plate-bound AF-2 or TDB (1 or 8 nmol/well) with solubilised LPS (100 ng/mL) or nigericin (10 μM) as a control and IL-1β was measured. Data represent the Mean ± SEM of three to six independent experiments (murine BMDMs) or three (human monocyte) experiments performed in triplicate. Statistical significance was calculated in comparison to iPrOH using 2-way ANOVA (Dunnett’s multiple comparison test), **** *P* ≤ 0.0001, *** *P* ≤ 0.001, ** *P* ≤ 0.01, * *P* ≤ 0.05.

To explore the mode of action of AF-2, we investigated the ability of AF-2 to induce cytokine and chemokine production using WT and Mincle^−/−^ BMDMs. The commonly used plate-bound assay was performed, whereby the AF-2 and TDB were first dissolved in an organic solvent, added to the bottom of a tissue culture plate, and the solvent then allowed to evaporate to dryness in a sterile hood. Cytokine and chemokine production was then measured by ELISA. AF-2 led to the production of IL-1β, IL-6, IL-23, MIP-2, TNF-α, IL-12p40 and IL-10 by WT BMDMs (Fig. 2a). Levels of IL-18 and IL-13 were too low to be detected. All responses were Mincle-dependent except for IL-1β production (*P* ≤ 0.001) and IL-12 production (*P* ≤ 0.05), with AF-2, but not TDB, leading to a significant increase in these cytokines in the absence of Mincle. We also measured the production of reactive oxygen species (ROS) following the stimulation of WT and Mincle^−/−^ BMDM with AF-2 and TDB. However, in the absence of priming, ROS production was not observed by either AF-2 or TDB (SI, Fig. 1).

The strong AF-2-mediated IL-1β response in the absence Mincle was unexpected and prompted us to reassess the ability of AF-2 to lead to IL-1β production by WT and Mincle^−/−^ BMDMs at two different glycolipid concentrations (1 and 8 nmol/well) and to compare these responses to those elicited by TDB and the NLRP3 inflammasome activator, nigericin [29]. WT and Mincle^−/−^ BMDMs produced IL-1β in response to AF-2 in a concentration-dependent manner, while TDB showed only Mincle-dependent IL-1β production with little change in the quantity of IL-1β being induced at both ligand concentrations (Fig. 2b). Like AF-2, nigericin led to a strong IL-1β response in both cell types. When AF-2 and TDB were added in PBS, the Mincle-dependent production of IL-1β was observed (Fig. 2c), thus highlighting how glycolipid presentation can affect the immune response [31–33]. We then assessed whether plate-coated AF-2 was able to lead to IL-1β production in human monocytes. In the absence of LPS priming, TDB was unable to induce detectable IL-1β production in human monocytes after 24 h (Fig. 2d). In contrast, at a concentration of 1 nmol/well, AF-2 induced IL-1β production by human monocytes, although this response was abrogated at higher concentrations of AF-2.

### Plate-coated AF-2 Induces Death of BMDMs in a Mincle-independent Manner

**Fig. 3:**
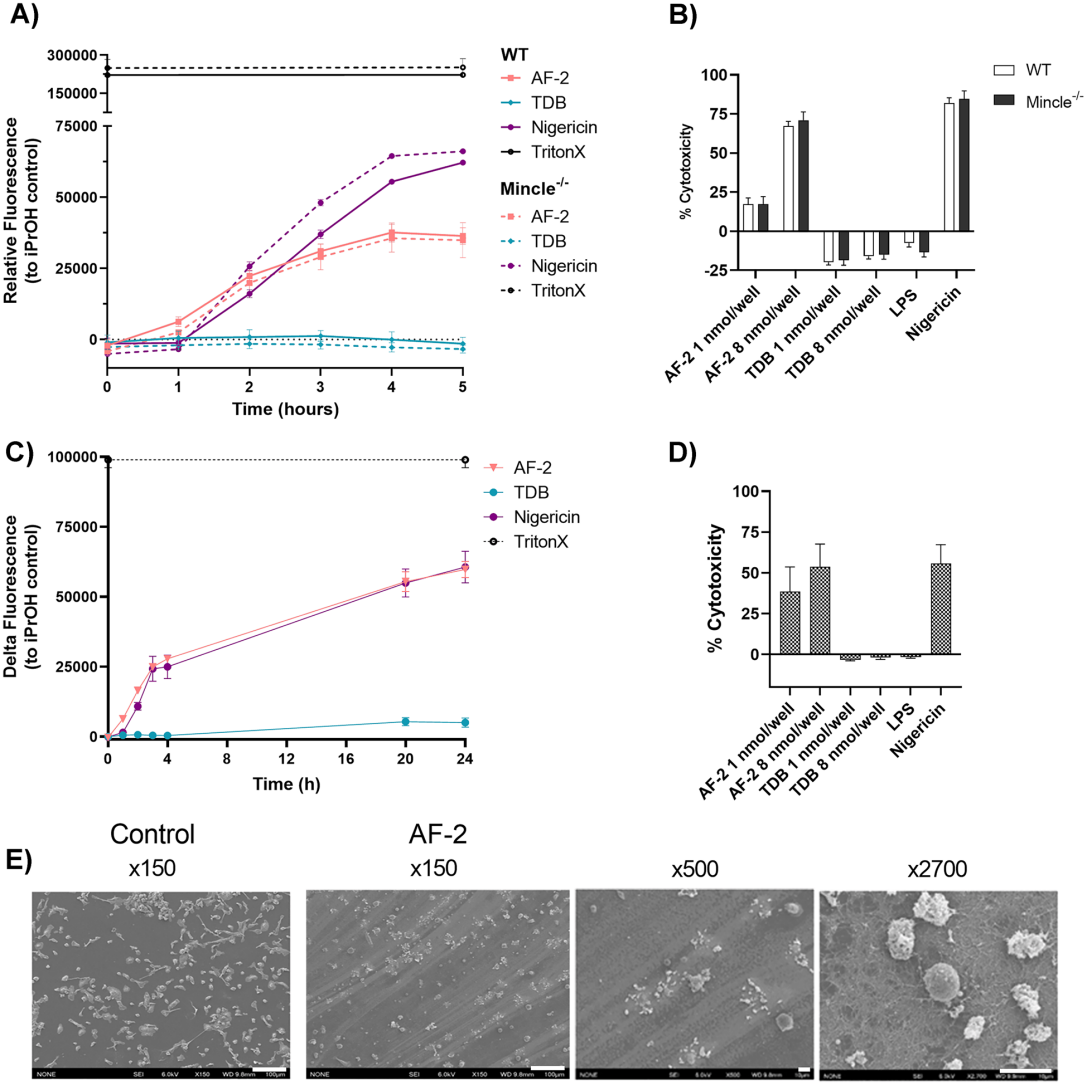
Plate-coated aryl-trehalose glycolipid, AF-2, induces murine BMDM and human monocyte death. **a** and **b** WT and Mincle^−/−^ GM-CSF BMDMs or **c** and **d** negatively enriched human monocytes were incubated in plates coated with AF-2 or TDB (8 nmol/well concentration in Sytox Green assay, 1 or 8 nmol/well concentration in LDH assay); solubilised LPS (100 ng/mL), nigericin (10 μM), or Triton X (1%). **a** and **c** Fluorescence of Sytox Green (1 μM) was measured over time; results are calculated relative to iPrOH control fluorescence. **b** and **d** LDH release was measured at 24 h from the supernatant. Data represent the Mean ± SEM of **a** a representative experiment repeated three times in triplicate, **b**-**d** three independent experiments performed in triplicate. GM-CSF BMDMs from WT mice were seeded on **e** cover slips alone or on coverslips coated overnight with AF-2 (8 nmol/cover slip) and imaged by scanning electron microscope (SEM) at ×150, ×500, and ×2,700 magnification. Scale bar: 100 μm for ×150 magnification and 10 µm for ×500 and ×2700 magnifications.

The production of IL-1β can be associated with cell death [34]. Accordingly, the ability of plate-coated AF-2 to induce cell death was investigated using the Sytox Green assay [35]. In this assay, detection of a cell membrane impermeable nucleic acid stain is proportionally related to the cell nuclear contents, and thus, simultaneously provides a measure of membrane integrity and cell death. For murine WT and Mincle^−/−^ BMDMs, AF-2, but not TDB, induced cell death, with this reaching a plateau after *ca.* 4–6 h when using 8 nmol/well of glycolipid (Fig. 3a, SI Fig. 2a). Cell death by AF-2 was dose dependent, with an increasing concentration of AF-2 (0.01, 0.1, 1, 8 10 nmol/well) increasing membrane degradation, as measured at the 24 h time point in WT BMDMs (SI, Fig. 2b). AF-2 also induced cell death in murine RAW264.7 cells (SI, Fig. 2c).

Further evidence for cell death and a loss of membrane integrity upon the treatment of BMDMs with AF-2 was observed by measuring the release of cytoplasmic lactate dehydrogenase (LDH) [36]. AF-2 led to a concentration dependent leakage of intracellular LDH for both WT and Mincle^−/−^ murine BMDMs, with cell death being enhanced when using a higher concentration of AF-2 (Fig. 3b). No extracellular LDH was detected upon the treatment of WT and Mincle^−/−^ BMDMs with TDB at either glycolipid concentration. Rather, TDB stimulation impaired LDH leakage from WT and Mincle^−/−^ BMDMs resulting in a negative percentage of cytotoxicity compared to the isopropanol control. Cell death was also observed when treating human monocytes with AF-2, as indicated by the Sytox Green assay (Fig. 3c) and by LDH release (Fig. 3d). Stimulation of human monocytes with TDB did not result in cell death. Scanning electron microscopy (SEM) also revealed that BMDMs remained viable and were numerous after being seeded on coverslips overnight (Fig. 3e). Treating the plates with AF-2 led to striated ring-like deposits on the plate, and the BMDMs were observed to undergo cell death (Fig. 3e). TDB did not lead to any obvious striations when coated on the cover-slips. The cells treated with TDB were as abundant as the untreated control cells but were more highly adherent and extracellular membrane bound vesicles could be observed (SI, Fig. 3).

### Plate-coated AF-2 Induces Caspase 1-dependent Pyroptosis

**Fig. 4:**
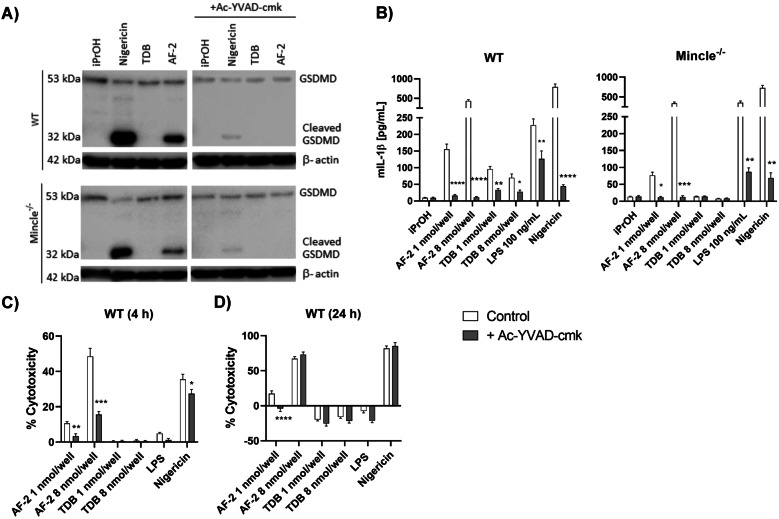
Aryl-trehalose glycolipid AF-2 induces Caspase-1 mediated induced pyroptotic cell death. WT and Mincle^−/−^ GM-CSF BMDMs were left untreated or pre-treated with Caspase-1 inhibitor Ac-YVAD-cmk (40 μM) before adding to plates coated with AF-2 or TDB (8 nmol/well concentration for Western Blot; 1 or 8 nmol/well concentration for IL-1β ELISA and LDL assay), or stimulation with solubilised LPS (100 ng/mL), nigericin (10 μM). **a** After 4 h the cells were lysed and whole cell lysates analysed for GSDMD cleavage by Western Blot. Data are representative of an experiment repeated three times. **b** IL-1β was measured in the supernatant at 24 h by ELISA. **c** and **d** LDH release was measured after 4 h and 24 h from the supernatant. Results are calculated relative to iPrOH control fluorescence. **b**, **c** and **d** Data represent the Mean ± SEM of three experiments performed in triplicate. Statistical significance was calculated in comparison to control using Multiple t-test (Mann-Whitney), **** *P* ≤ 0.0001, *** *P* ≤ 0.001, ** *P* ≤ 0.01, * *P* ≤ 0.05.

We then sought to provide further insight into the mode of action of cell death elicited by plate-coated AF-2. A hallmark of pyroptosis is the cleavage of gasdermin D (GSDMD) [34, 37]. This leads to the formation of pores in the plasma membrane that allow for the release of cytoplasmic contents including IL-1β and LDH [34, 38]. The cleavage of GSDMD was investigated by Western Blot following the treatment of WT and Mincle^−/−^ BMDMs with AF-2 and nigericin (positive control) (Fig. 4a). In both cell types, AF-2 led to GSDMD cleavage, as did nigericin, while TDB did not. GSDMD is a substrate for Caspase-1 [39]. The addition of the Caspase-1 inhibitor Ac-YVAD-cmk prevented GSDMD cleavage in both cell types, thus confirming Caspase-1 mediated pyroptotic cell death. Caspase-1 cleaves pro-IL-β [39]. We observed that the production of IL-1β by AF-2 was also dependent on Caspase-1, with the addition of Ac-YVAD-cmk leading to a significant decrease in IL-1β production by WT and Mincle^−/−^ BMDMs (Fig. 4b). Caspase-1 inhibition prevented IL-1β production in response to TDB, which was consistent with the observation that the NOD-like receptor family pyrin domain containing 3 (NLRP3) inflammasome and Caspase-1 activation is required for TDB-mediated IL-1β production [12, 40]. The addition of Ac-YVAD-cmk to AF-2-treated BMDMs led to a significant decrease in LDH release at 4 h at both concentrations of AF-2 used, and a significant decrease in LDH release when using the lower concentration of AF-2 at 24 h for both WT and Mincle^−/−^ BMDMs (Fig. 4c, d, SI Fig. 4a, b). The addition of Ac-YVAD-cmk to wild type BMDMs also decreased AF-2-mediated cell death, as evidenced using the Sytox Green assay (SI Fig. 4c).

### Plate-coated AF-2 Induces NLRP3 Inflammasome-mediated Pyroptosis

**Fig. 5:**
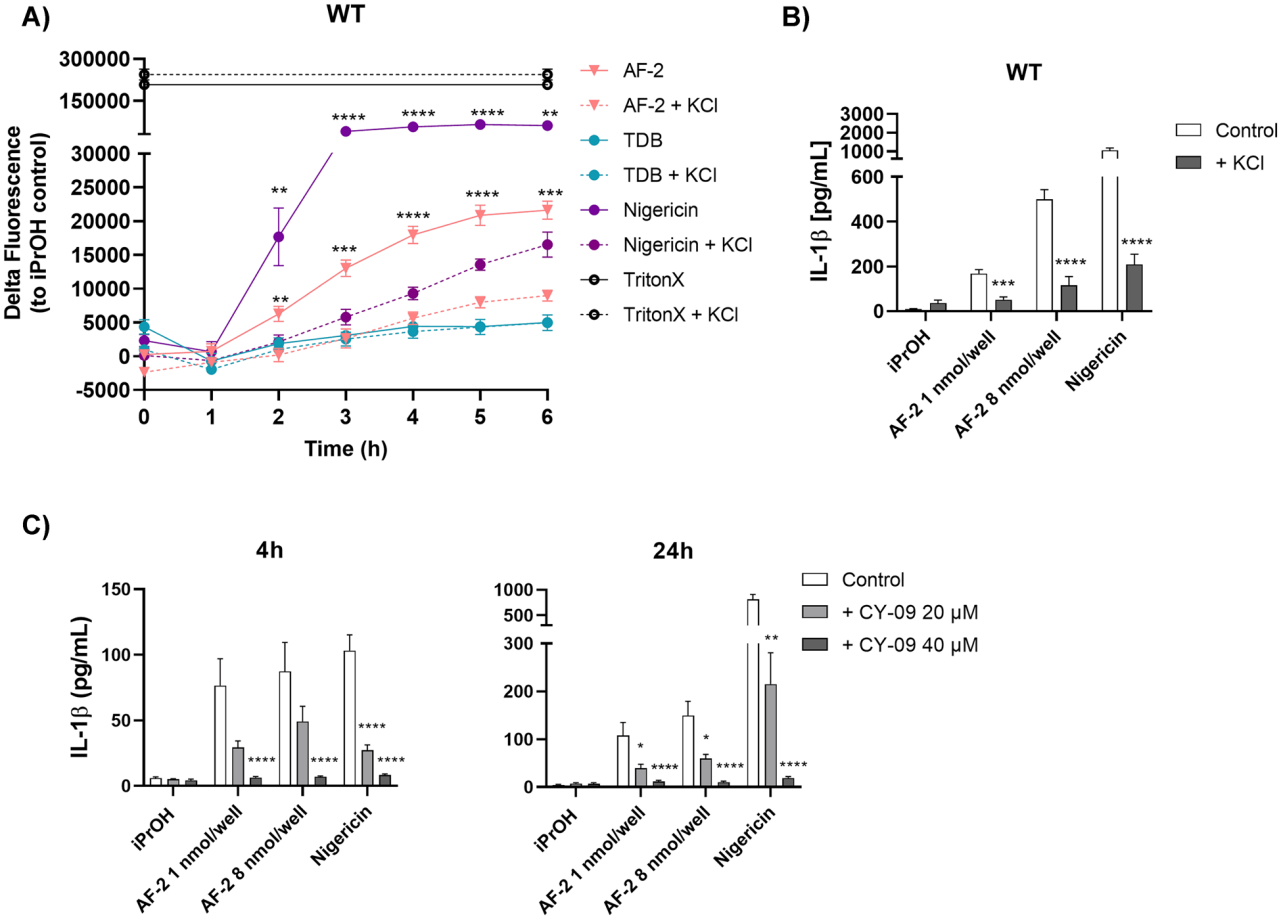
K^+^ efflux and NLRP3 inflammasomes are involved in AF-2 induces pyroptotic cell death. WT and Mincle^−/−^ GM-CSF BMDMs were left untreated or pre-treated with KCl (50 nm) or NLRP3 inflammasome inhibitor CY-09 (20 or 40 µM) before being added to plates coated with AF-2 or TDB (8 nmol/well concentration for Sytox Green assay; 1 or 8 nmol/well concentration for IL-1β ELISA), or stimulated with solubilised nigericin (10 μM), or Triton X (1%). **a** Sytox Green fluorescence intensity (1 μM) was measured over time; results are calculated relative to iPrOH control fluorescence. **b** IL-1β was measured in the supernatant at 24 h by ELISA or **c** at 4 and 24 h by ELISA. **a** Data represent the Mean ± SEM of a representative experiment repeated three times. **b**, **c** Data represent the Mean ± SEM of three independent experiments performed in triplicate. Statistical significance was calculated in comparison to untreated control using Multiple t-test (Mann–Whitney), **** *P* ≤ 0.0001, *** *P* ≤ 0.001, * *P* ≤ 0.05.

**Fig. 6:**
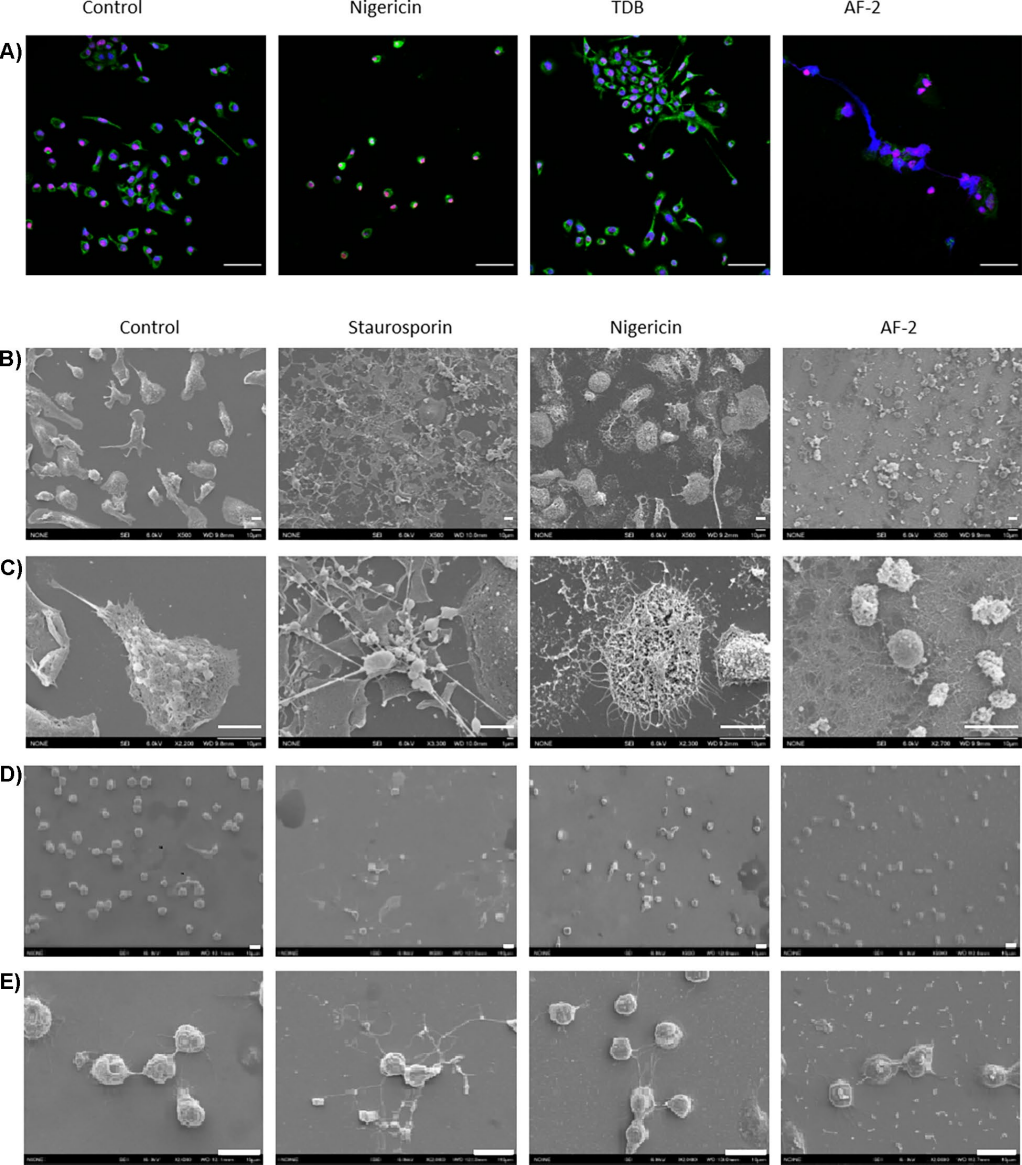
Morphological changes induced by AF-2 are indicative of lytic cell death. **a** GM-CSF BMDMs from WT mice were seeded on cover slips coated with AF-2 or TDB (8 nmol/cover slip) or stimulated with nigericin (10 μM) and imaged by confocal microscopy after 4 h. Merged images of MPO (green), Histone 4 (red), and DAPI (blue). Scale bar: 50 μm. Scanning electron microscope (SEM) images of GM-CSF BMDMs and at **b** low (× 500) and **c** high (× 2,200–3,300) magnification following treatment with AF-2 (24 h, nmol/slide), staurosporine (5 h, 1 μm) as a control for apoptosis, or nigericin (1 h, 10 μM) as a control for pyroptosis. Scale bar: 10 μM). RAW264.7 cells were primed with LPS (1 μg/mL) and seeded on conductive silicon wafers coated with AF-2 (24 h, 8 nmol/slide) or stimulated with staurosporine (5 h, 1 μM) or nigericin (10 μM). Scanning electron cyro microscope (CyroSEM) images at **d** low (× 500) and **e** high (× 2,200–3,300) magnification. Scale bar: 10 μm.

Caspase-1 containing inflammasomes consist of NLR inflammasomes, NLRP3 and NLRC4, and the absent in melanoma 2 (AIM2) inflammasomes [41, 42], whereby the latter binds to cytosolic dsDNA [41]. NLRP3 and NLRC4 are differentially affected by K^+^ efflux, with the concentration of extracellular K^+^ required to inhibit NLRC4 being reported to be higher (> 90 mM KCl) than that required for NLRP3 inhibition [43]. Using 50 mM KCl, we observed a decrease in the AF-2- and nigericin-mediated deaths of WT (Fig. 5a) and Mincle^−/−^ BMDMs (SI Fig. 5a), but there was no significant effect on TDB treated cells. The addition of extracellular KCl decreased AF-2 mediated LDH release from both WT and Mincle^−/−^ BMDMs (SI Fig. 5b, c), and led to the abolition of IL-1β production by WT BMDMs after stimulation with AF-2 (**3**) and nigericin (Fig. 5b, SI Fig. 5d). To confirm the role of the NLRP3 inflammasome in AF-2-mediated cell death, we added titrated quantities of the NLRP3 inflammasome inhibitor CY-09 [44] to BMDMs treated with AF-2 and measured IL-1β production at 4 h and 24 h (Fig. 6c). A significant concentration dependent decrease in IL-1β was observed for both concentrations of AF-2 at both time points following the addition of CY-09. A similar response was observed for nigericin (positive control).

### Plate-coated AF-2 Leads to Morphological Changes in Macrophages Indicative of Pyroptosis

Pyroptosis is morphologically and mechanistically distinct from other forms of cell death [34, 45]. AF-2 stimulation caused cell swelling and the spillage of intracellular material that is characteristic of pyroptotic cell death [34, 45], as indicated by DAPI (double stranded DNA) and Histone 4 (chromatin) staining imaged by confocal microscopy (Fig. 6a). In contrast, in control and TDB treated cells a clear distinction between nuclear and cytoplasmic contents (MPO lysosomal stain) was observed, with TDB-treated cells exhibiting cell clustering. Cells treated with nigericin dramatically decreased in size and showed some membrane disruption. SEM of untreated control BMDMs showed intact adherent cells, while staurosporine treated cells revealed classic apoptotic bodies, and treatment with nigericin revealed corpses of pyroptotic cells with a ‘fried egg’ morphology (Fig. 6b, c) [46]. Treatment with AF-2 induced the formation of large networks of cellular connections and smaller cells with cellular blebs. Similar findings were observed *via* scanning electron cryomicroscopy (CryoSEM) of RAW264.7 cells treated with AF-2, staurosporine, or nigericin (Fig. 6d, e). Treatment with AF-2 led to a reduced number of adherent cells, a reduction in cell size, and the expulsion of intracellular material, which was not membrane bound.

## DISCUSSION

α,α′-Trehalose 6,6′-glycolipids exhibit a variety of immunodulatory properties [1]. These include TDM being a key virulence factor of *M. tuberculosis* [4] and the use of TDB as a vaccine adjuvant for both preventative and therapeutic vaccines [2]. In addition to the effect of their molecular structure on the ensuing immunological response [2], the physical presentation of α,α′-trehalose 6,6′-glycolipids can influence their immunological properties though mechanisms that are poorly understood. Phosphoproteome analysis has also revealed that plate-bound TDB can induce the phosphorylation of proteins in a Mincle-dependent and independent manner [47]. However, of the multitude of α,α′-trehalose 6,6′-glycolipids tested for their inflammatory properties, none have been shown to elicit Mincle-independent cytokine production. Accordingly, the Mincle-independent production of IL-1β following the stimulation of BMDMs with plate-bound AF-2 was highly unusual.

Previously, we determined that AF-2 is a promising Mincle-mediated adjuvant [25]. AF-2 activated NFAT-GFP reporter cells expressing murine or human Mincle, and WT BMDMs [25]. Insofar, we initially hypothesised that the expression of cytokines and chemokines by AF-2-stimulated BMDMs required activation of the Mincle-mediated FcRγ-Syk-Card9-Bcl10-Malt1 signalling axis [7–9]. Our studies herein demonstrated the Mincle-dependent production of cytokines (TNF-α, IL-6, IL-10, IL-23, IL-12p40) and chemokine (MIP-2) following the treatment of BMDMs with AF-2. However, using Mincle^−/−^ BMDMs, the Mincle-independent release of IL-1β was demonstrated by plate-bound AF-2. Some IL-12 was also observed following the treatment of Mincle^−/−^ BMDMs with plate-bound AF-2. IL-1β induces human monocyte-derived dendritic cells to produce IL-12 [48], and it is therefore possible that IL-1β can also induce BMDMs to produce IL-12. In the absence of priming, human monocytes treated with lower concentrations of AF-2, but not TDB, also led to IL-1β production.

The strong Mincle-independent production of IL-1β by plate-bound AF-2 prompted us to consider cell death as a possible cause. To this end, we treated WT and Mincle^−/−^ BMDMs, human monocytes, and RAW264.7 cells with plate-coated AF-2 and assessed cell viability using the Sytox Green Assay [35] and by measuring LDH release [36]. In all instances, lytic cell death was observed, with the percentage of cell death increasing over time before reaching a plateau. Cell death also increased with increasing concentration of AF-2, and the increase in cell death corresponded to an increase in IL-1β production by WT and Mincle^−/−^ BMDMs. In contrast, TDB did not induce lytic cell death and rather appeared to lead to a modest enhancement in cell viability. This observation was in agreement with earlier studies by Lang and co-workers where the Mincle-independent response to plate-coated TDM was associated with macrophage proliferation and survival [47].

Further insight into the mechanism of cell death by AF-2 was determined by examining the morphology of BMDM and RAW264.7 cells by confocal microscopy and SEM following treatment with AF-2 or TDB. The observed spillage of intracellular material and networks of cellular connections supported AF-2-mediated lytic cell death, and could be indicative of programmed cell death pathways such as pyroptosis or necroptosis, but not apoptosis, since the latter is characterised by the release of apoptotic bodies [34, 45, 46]. In contrast, the treatment of BMDM and RAW264.7 cells with TDB did not lead to cell membrane rupture, but resulted in cells that were more adherent, which is suggestive of an activated macrophage phenotype.

Unequivocal evidence for AF-2-mediated pyroptosis was obtained *via* cleavage of gasdermin D (GSDMD) for both WT and Mincle^−/−^ BMDMs upon treatment with AF-2, but not TDB. The addition of the caspase-1 inhibitor Ac-YVAD-cmk prevented GSDMD cleavage and led to a significant reduction in IL-1β production in both cell types, therefore providing further evidence for caspase-1-mediated pyroptosis. The inhibition of caspase-1 also lead to a reduction in cell death at 4 h when using both concentrations of AF-2, and at 24 h when using the 1 nmol/well concentration of AF-2. Although the inhibition of Capase-1 did not abolish cell death as measured by the LDH assay at the 48 h time point when using a higher concentration of AF-2, it is known that inhibiting the dominant molecular route of cell death, including GSDMD-dependent pyroptosis, can promote alternative cell death programs [49, 50]. Insofar, it is likely that when added at high concentrations, AF-2 “stressed” the cell sufficiently to activate an alternative cell death pathway. In addition, the administration of 50 mM of KCl reduced plate-bound AF-2-mediated cell death and IL-1β production by BMDMs, while NLRP3 inflammasome inhibition using CY-09 inhibited the production of IL-1β by AF-2-treated BMDMs in a concentration-dependent manner. Taken together, this data demonstrated AF-2-induced pyroptotic cell death by Caspase-1 dependent NLRP3 inflammasome-mediated activation.

The ability of plate-coated AF-2 to lead to pyroptotic cell death was surprising and prompted us to explore whether glycolipid presentation influenced the immune response to AF-2. AF-2 led to striated ring-like deposits on an electron microscopy plate, which was in contrast to TDB where no such uneven plate-coating, or cell death, was observed. When using plate-coated assays, macrophage and monocytes settle to the bottom of the wells and the observed immunological activity is thus primarily attributed to the immune cells interacting with the plate-bound ligands. In contrast, when added in solution, AF-2 led to Mincle-dependent IL-1β production.

Further studies are required to determine why the presentation of α,α′-trehalose glycolipids may influence their immunomodulatory properties [31–33]. In previous studies, water-soluble α,α′-trehalose 6,6′-glycolipids have been shown to signal through Mincle but were unable to activate phagocytic pathways and the NLRP3 inflammasome [16]. In contrast, particulate materials, such silica and alum, can induce pyroptosis-mediated cell death *via* phagocytosis and lysosomal damage [41, 51]. Thus, if activation of the inflammasome is a continuum [52], perhaps plate-coated AF-2 is ‘ideally’ aggregated to augment phagocytosis, lysosome cleavage, and the release of particulates into the cytoplasm, thereby increasing the inflammatory insult to the cell and causing the cell to commit to cell death. Whether inflammasome activation occurs in a one-step process, as was observed for THP-1 cells treated with silica [53], or *via* a two-step process, which would require AF-2 to bind to a different receptor(s) or effect the enrichment of different transcription factors [54, 55] to provide signal 1 in Mincle-independent manner, is unknown. Notwithstanding, the presentation of AF-2 needs to be taken into account when considering how to administer the ligand as an adjuvant for vaccinations against infectious disease [2], where cell-death may be less desirable, or for application in anti-cancer therapies, where pyroptosis-mediated tumor clearance is a promising new approach [56–58].

## CONCLUSION

The α,α′-trehalose 6,6′-glycolipid AF-2 signals through Mincle to lead to the induction of an inflammatory immune response by macrophages. When in soluble form, this response is abrogated in the absence of Mincle. However, when AF-2 is plate-bound the Mincle-independent production of IL-1β is observed and the cells commit to cell death *via* caspase-1-dependent NLRP3 inflammasome-mediated pyroptosis. High levels of IL-1β are also observed following the treatment of human monocytes with AF-2. Insofar, the immunological response to AF-2 differs depending on its mode of presentation. This sets it apart from other α,α′-trehalose 6,6′-glycolipids that have been tested in the conventional plate-bound assay. Our finding also highlights the need to investigate the physical presentation of α,α′-trehalose 6,6′-glycolipids so that the adjuvanticity of these compounds can be best optimised for application in either infectious disease or anti-cancer vaccines.

## Supplementary Information

Below is the link to the electronic supplementary material.

Supplementary file1 (JPG 67 KB)

Supplementary file2 (JPG 30 KB)

Supplementary file3 (PDF 511 KB)

Supplementary file4 (JPG 37 KB)

Supplementary file5 (JPG 39 KB)

## Data Availability

Not applicable.
