# Supplementary figures and images for: Aryl-functionalised α,α′-Trehalose 6,6′-Glycolipid Induces Mincle-independent Pyroptotic Cell Death
Source: Inflammation. 2023 May 4;46(4):1365–80. doi: 10.1007/s10753-023-01814-5 (PMC10359228; doi:10.1007/s10753-023-01814-5)

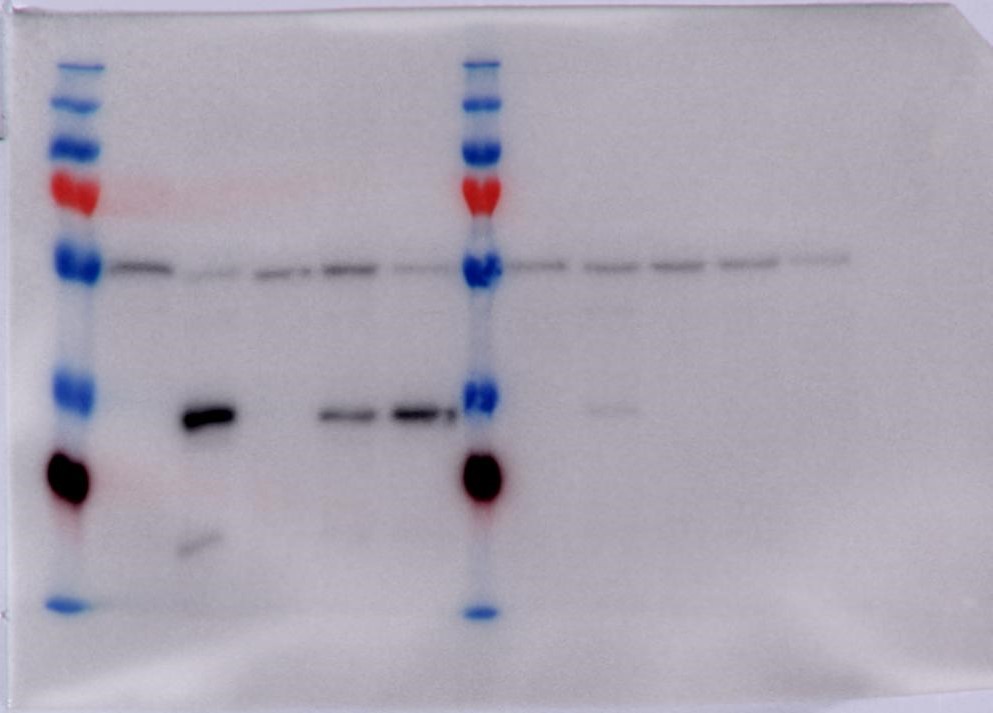

Supplement: Supplementary file 1 — Supplementary file1 (JPG 67 KB) [file 10753_2023_1814_MOESM1_ESM.jpg]

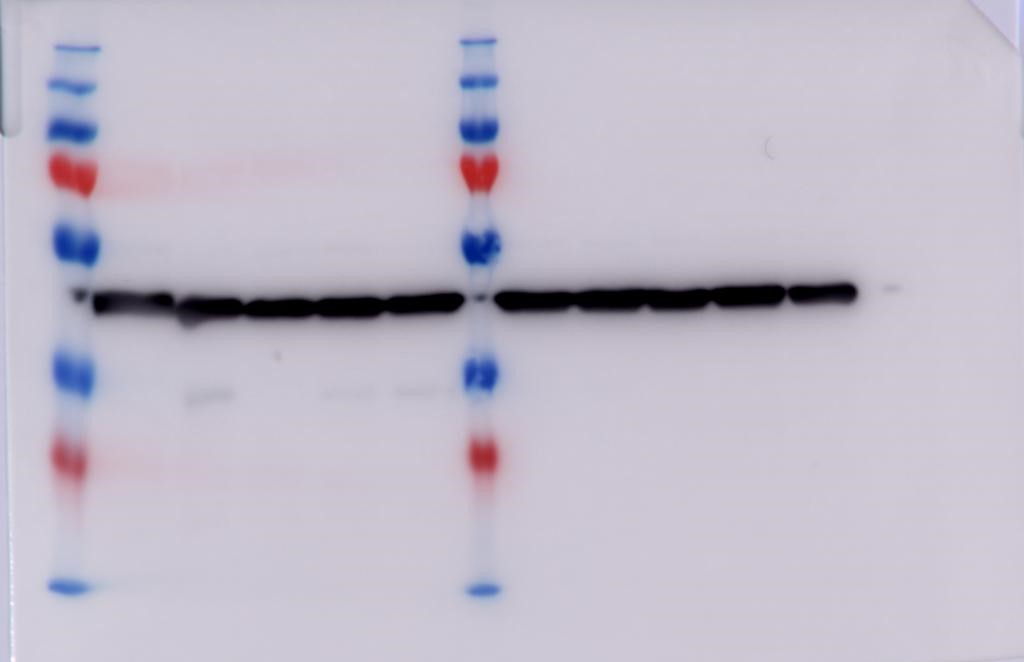

Supplement: Supplementary file 2 — Supplementary file2 (JPG 30 KB) [file 10753_2023_1814_MOESM2_ESM.jpg]

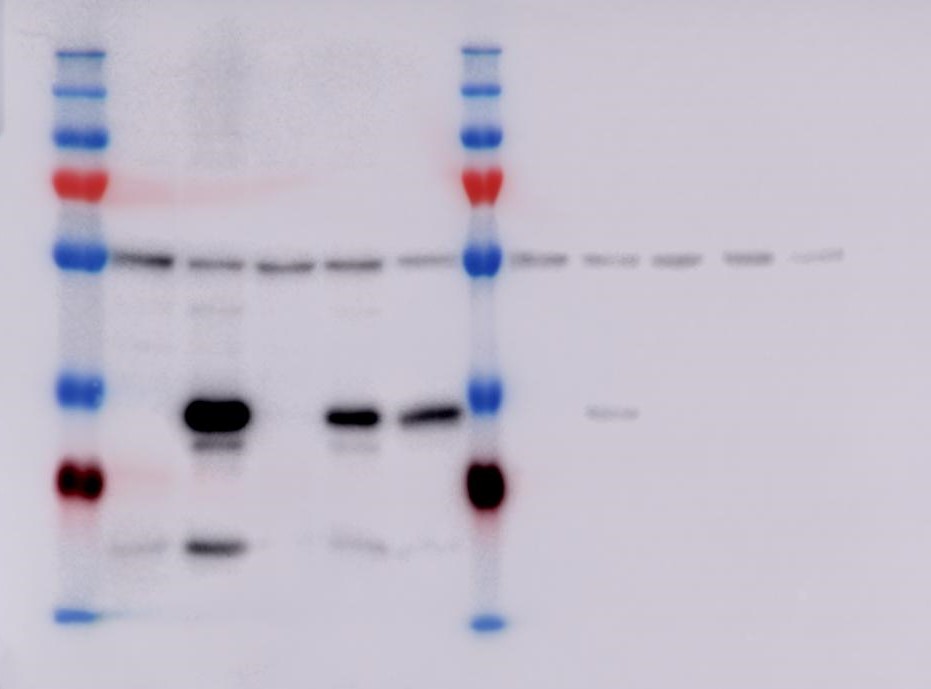

Supplement: Supplementary file 4 — Supplementary file4 (JPG 37 KB) [file 10753_2023_1814_MOESM4_ESM.jpg]

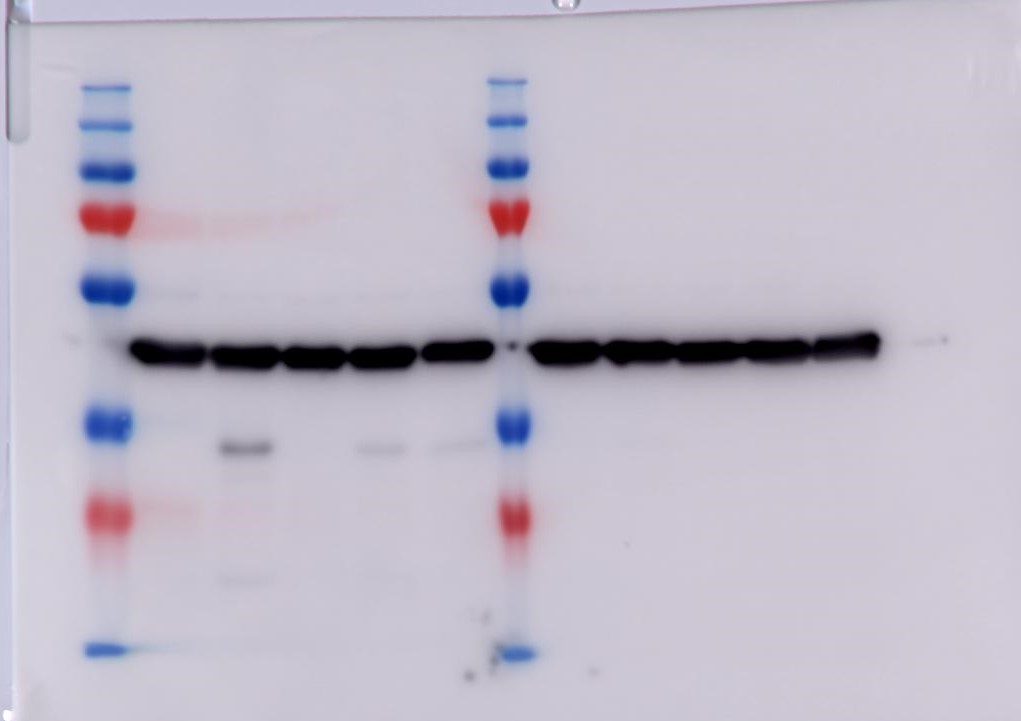

Supplement: Supplementary file 5 — Supplementary file5 (JPG 39 KB) [file 10753_2023_1814_MOESM5_ESM.jpg]
