# Supplementary material for: Aryl-functionalised α,α′-Trehalose 6,6′-Glycolipid Induces Mincle-independent Pyroptotic Cell Death
Source: Inflammation. 2023 May 4;46(4):1365–80. doi: 10.1007/s10753-023-01814-5 (PMC10359228; doi:10.1007/s10753-023-01814-5)
Supplement: Supplementary file 3 — Supplementary file3 (PDF 511 KB) [file 10753_2023_1814_MOESM3_ESM.pdf]

~ SUPPORTING INFORMATION ~

Aryl-functionalised α,α′-trehalose 6,6′-glycolipid induces Mincle-independent pyroptotic cell death

Kristel Kodar,[a,b] Emma M. Dangerfield,[a,b] Amy J. Foster,[a,b] Devlin Forsythe,[b,c] Shigenari Ishizuka,[d,e] Melanie J. McConnell,[b,c] Sho Yamasaki,[d,e,f,g] Mattie S. M. Timmer,[a,b,*] Bridget L. Stocker [a,b,*]

[a] School of Chemical and Physical Sciences, Victoria University of Wellington, PO Box 600 Wellington, New Zealand
[b] Centre for Biodiscovery, Victoria University of Wellington, PO Box 600, Wellington, New Zealand
[c] School of Biological Sciences, PO Box 600, Wellington, New Zealand
[d] Department of Molecular Immunology, Research Institute for Microbial Diseases, Osaka University, Suita, Osaka, Japan
[e] Laboratory of Molecular Immunology, Immunology Frontier Research Center, Osaka University, Suita, Osaka, Japan
[f] Division of Molecular Immunology, Medical Institute of Bioregulation, Kyushu University, Fukuoka, Fukuoka, Japan
[g] Division of Molecular Immunology, Medical Mycology Research Center, Chiba University, Chiba, Japan

*Corresponding authors: Tel.: +64 4 463 6529; fax: +64 4 4 463 5241
E-mail address: bridget.stocker@vuw.ac.nz; mattie.timmer@vuw.ac.nz

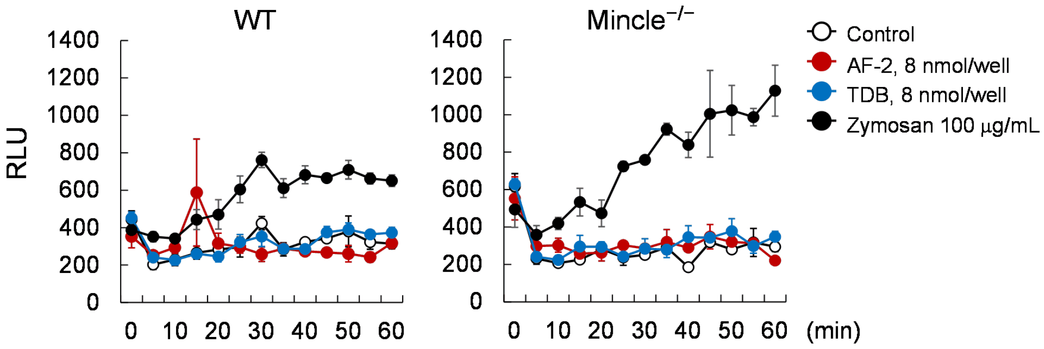

SI Figure 1: **AF-2 did not induce ROS production in un-primed GM-CSF BMDMs.** Bone marrow cells from WT and Mincle$^{-/-}$ mice were cultured in 10 % FCS/RPMI supplemented with 50 ng/mL GM-CSF (BioLegend) for eight days. Cells were detached using 1 mM EDTA-supplemented PBS and added into 96-well plates coated with 8 nmol/well of AF-2 or TDB, or 100 mg/mL zymosan (SIGMA), in the presence of 300 μM luminol (Nacalai tesque) for 60 min. Luminescence was quantified by POWERSCAN HT (DS Pharma Biomedical). Data are presented as mean ± SE of triplicate assays.

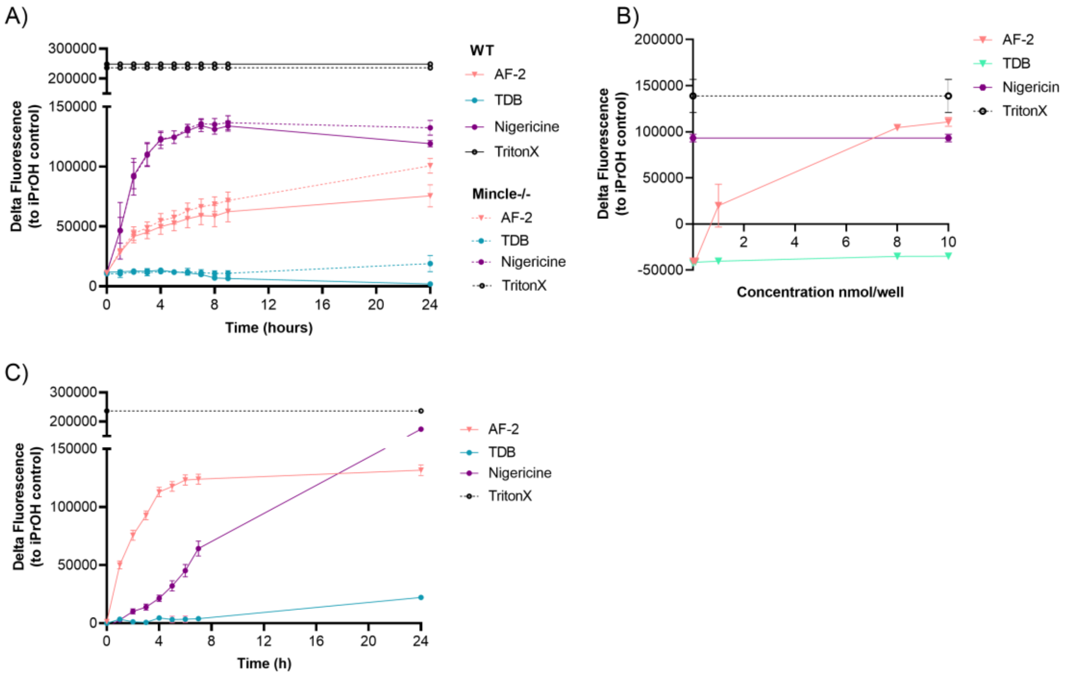

SI Figure 2. The kinetics of AF-2 induced cell death. A) Wild-type (WT) and Mincle$^{-/-}$ GM-CSF BMDMs or C) 5 ng/mL LPS primed RAW264.7 cells were incubated in plates coated with AF-2 or TDB (8 nmol/well), solubilised nigericin (10 µM), or Triton X (1%). Fluorescence of Sytox Green (1 µM) was measured over time; results are calculated relative to iPrOH control fluorescence. B) C57BL/6 GM-CSF BMDMs were incubated in plates coated with various concentrations of AF-2 or TDB (0.01, 0.1, 1, 8 and 10 nmol/well), solubilised nigericin (10 µM), or Triton X (1%), and SytoxGreen fluorescence was measured at 24 h. Data represents the Mean ± SEM of a representative experiment performed three times in triplicate.

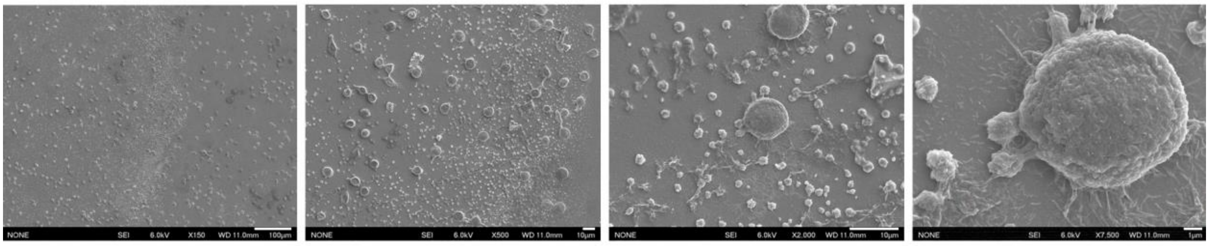

SI Figure 3. Morphological changes induced by TDB. SEM of RAW264.7 cells stimulated with TDB. RAW264.7 cells were primed with LPS (1 µg/mL) and seeded on conductive silicon wafers coated with TDB (4 h, 8 nmol/slide). Scanning electron cyromicroscope (CyroSEM) images were taken at four magnifications (x 150, x500, x2,000, x7,500). The white scale bar indicates 100, 10, 10 or 1 µm for the x 150, x500, x2,000, x7,500 magnifications, respectively.

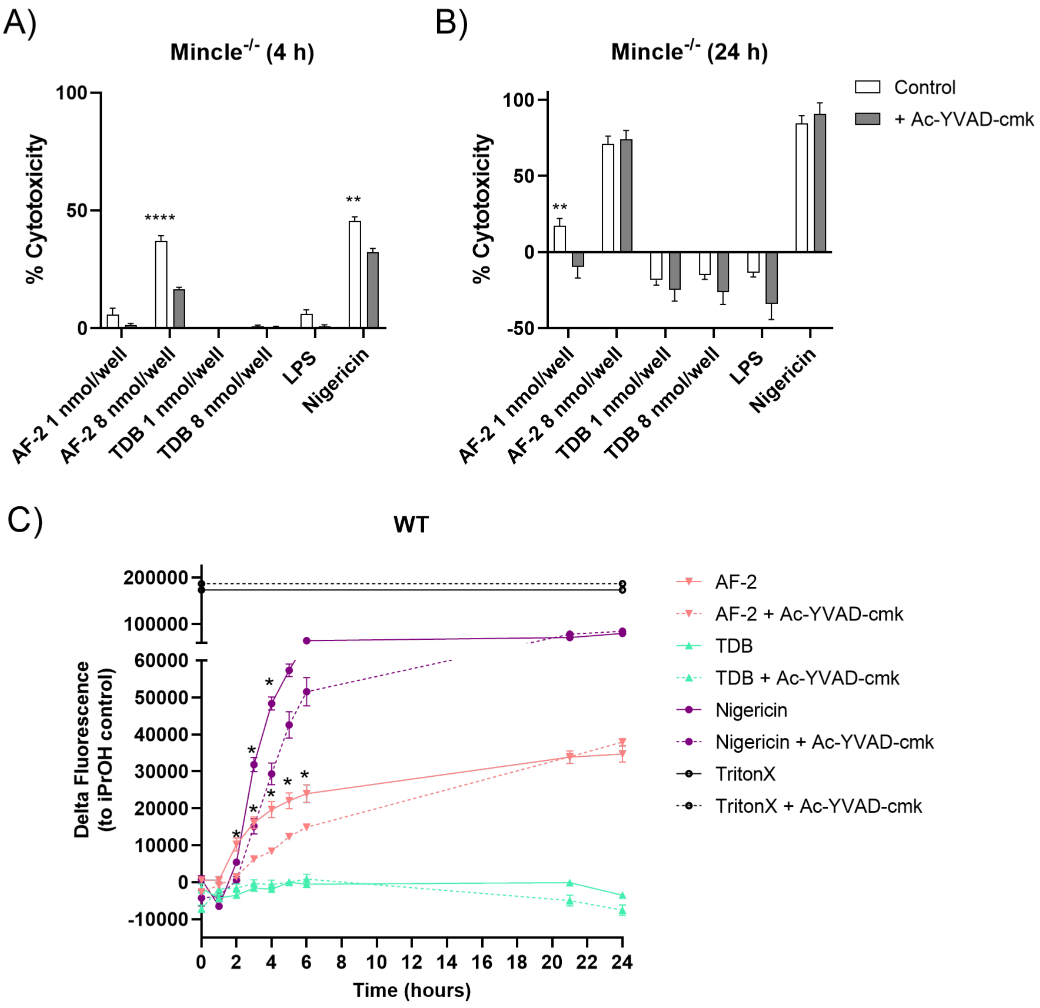

SI Figure 4. The effect of Caspase-1 inhibition on AF-2 induced cell death. A) Wild-type (WT) and Mincle$^{-/-}$ GM-CSF BMDMs were left untreated or pre-treated with Caspase-1 inhibitor AcYVAD-cmk (40 µM) before adding to plates coated with AF-2 or TDB (8 nmol/well concentration for Sytox Green assay; 1 or 8 nmol/well for LDH assay), or stimulated with solubilised LPS (100 ng/mL), nigericin (10 µM) or TritonX (1%). LDH release was measured at A) 4 h and B) 24 h from the supernatant. Data represent the Mean ± SEM of three independent experiments performed in triplicate. C) Fluorescence of Sytox Green (1 µM) was measured over time; results are calculated relative to iPrOH control fluorescence. Data represents the Mean ± SEM of a representative experiment performed three times in triplicate. Statistical significance was calculated in comparison to iPrOH using Multiple t-test, **** $P \leq 0.0001$, ** $P \leq 0.01$, * $P \leq 0.05$.

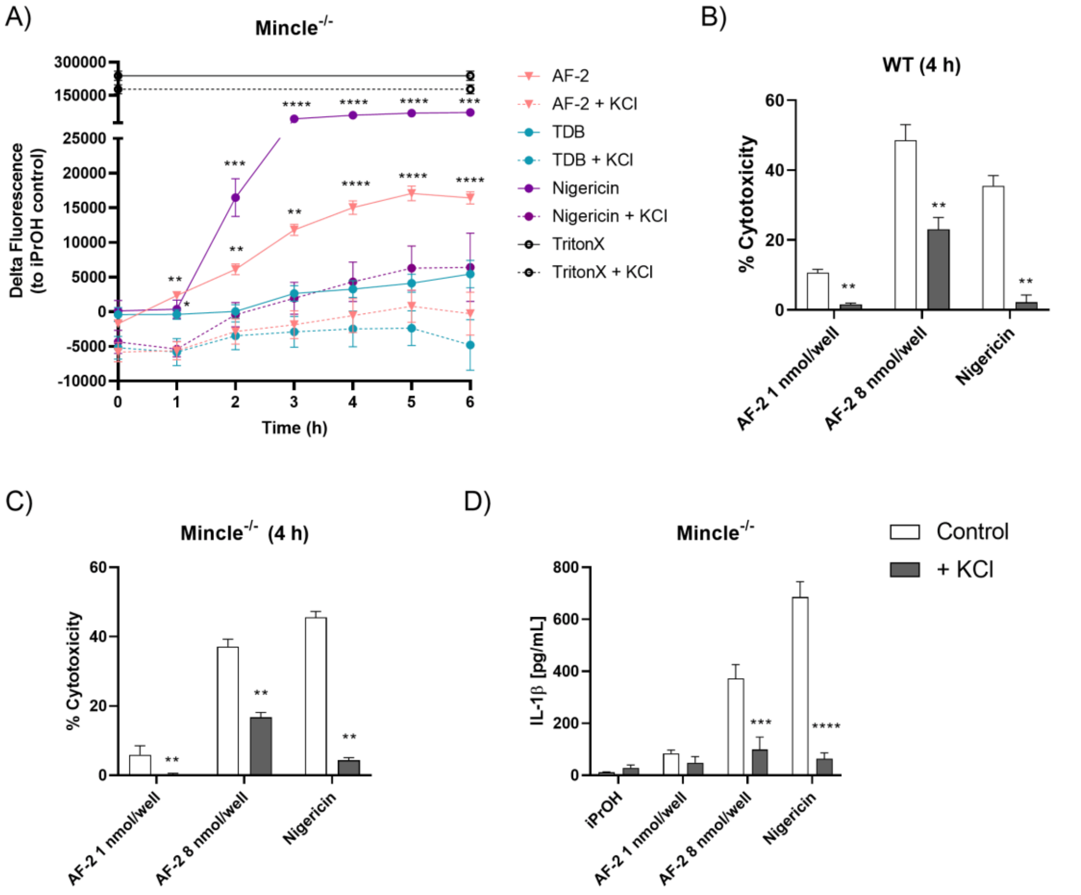

SI Figure 5. AF-2 induced pyroptosis is also dependent on K^+ efflux.

Wild-type (WT) and Mincle$^{-/-}$ GM-CSF BMDMs were pre-treated with KCl (50mM) before adding to plates coated with AF-2 (8 nmol/well concentration for SytoxGreen assay; 1 or 8 nmol/well for LDH assay), or stimulated with nigericin (10 μM) or TritonX (1%). A) Fluorescence of Sytox Green (1 μM) was measured over time; results are calculated relative to iPrOH control fluorescence. LDH release was measured at 4 h and in B) WT and C) Mincle$^{-/-}$ mice from the supernatant. D) IL-1β was measured from the supernatant at 24 h by ELISA. Data represent the Mean ± SEM of A) a representative experiment performed three times in triplicate, B-D) three experiments performed in triplicate. Statistical significance was calculated in comparison to control using Multiple t-test, **** $P \leq 0.0001$, *** $P \leq 0.001$, ** $P \leq 0.01$, * $P \leq 0.05$.

*****
